# Urethral Hairballs as a Long-Term Complication of Hypospadias Repair: Two Case Reports

**DOI:** 10.1155/2012/769706

**Published:** 2012-08-08

**Authors:** Spyridon Kampantais, Charalampos Dimitriadis, Leonidas Laskaridis, Ioannis Perdikis, Petros Kirtsis, Chrysovalantis Toutziaris

**Affiliations:** ^1^A' Urologic Department of Aristotle, University of Thessaloniki, 54635 Thessaloniki, Greece; ^2^Urologic Department of General Hospital of Drama, 66100 Drama, Greece

## Abstract

Many times hair-bearing urethral grafts have been used inadvertently in the treatment of hypospadias. This can be accompanied with numerous troublesome long-term complications such as formation of stones, diverticula, and hairballs. We report two cases of men with a history of hypospadias repair being affected by such complications. We also discuss about their management and the effect of thioglycolic acid instillation to stop hair growth in the urethra mucosa in the second case.

## 1. Introduction

Hypospadias represents one of the most challenging problems in pediatric urology. It is usually accompanied by many kinds of complications such as urethrocutaneous fistula, stricture and neourethral diverticulum [1]. One uncommon but distressing problem that can be observed is urethral hair growth and hairball formation, when hair-bearing skin is included in the reconstruction of the urethra [2]. We reproduce our experience with two men presenting lower urinary tract symptoms due to urethral hairballs. The first was treated by surgical removal while the second was initially treated with urethroscopic removal of the hairs, followed by instillation of depilating agents to prevent their regrowth. 

## 2. Case 1

**Figure 1:**
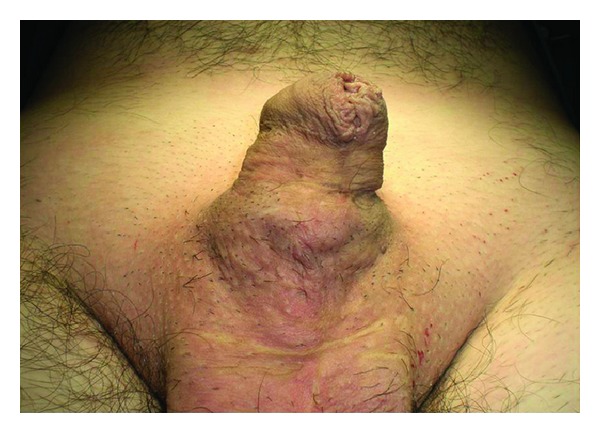
Case 1: Inspection revealing protrusion in the ventral side of the penile shaft.

A 34-year-old male presented with a history of a weak urinary stream, a mild penile pain, and a progressive swelling on the ventral side of his penis. He had undergone penile hypospadias repair at 3 years of age. Local examination revealed induration and protrusion in the middle underside of the penile shaft (Figure 1). Urethroscopy revealed an urethral hairball within a diverticulum in the penile urethra. The remaining length of the urethra did not present any abnormalities. 

**Figure 2:**
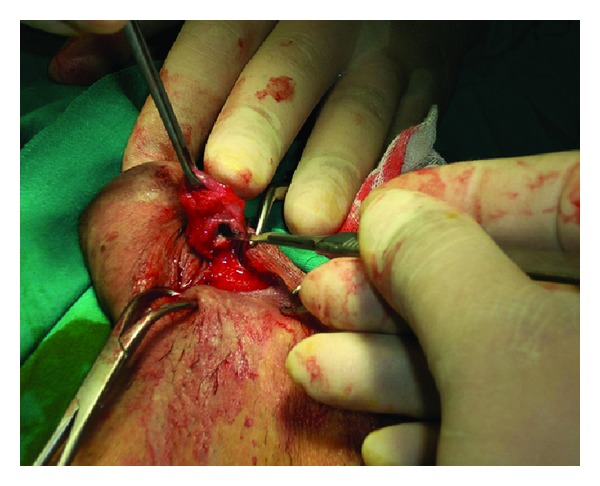
Case 1: surgical dissection of the urethral diverticulum.

**Figure 3:**
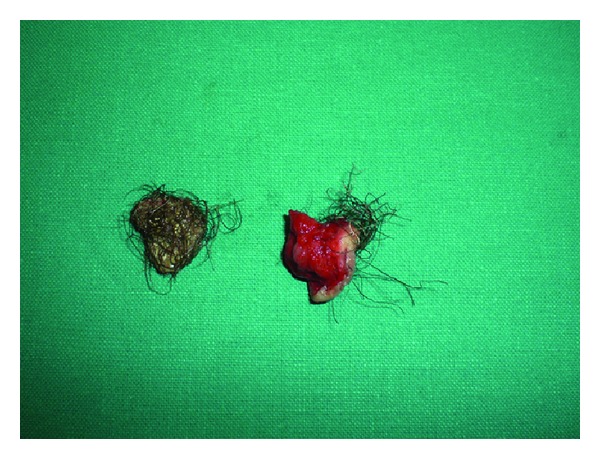
Case 1: removed diverticulum with stone and hairs.

After informed consent, the patient underwent surgical exploration. A 2 cm incision was made in the midline raphe of the penile shaft over the swelling. Careful dissection exposed a urethral diverticulum 1.5 cm in diameter (Figure 2). It was firmly attached to urethra containing a stone and multiple hairs (Figure 3). Excision of the diverticulum was performed by blunt and sharp dissection, and the urethral defect was closed transversally in two layers over the urethral Foley catheter. There were no complications postoperatively. The catheter was removed on the seventh postoperative day, and the patient was able to void normally. One year later the patient continues to report normal micturition.

## 3. Case 2

A 42-year-old man visited our hospital complaining of lower urinary tract symptoms since three months. His urological history was significant for scrotal hypospadias. He had undergone multi-staged urethroplasty in his childhood at ages 4 and 7 years old. Clinical examination revealed hairs protruding from the external urethral meatus and a palpable small mobile nodule in his penoscrotal junction. 

**Figure 4:**
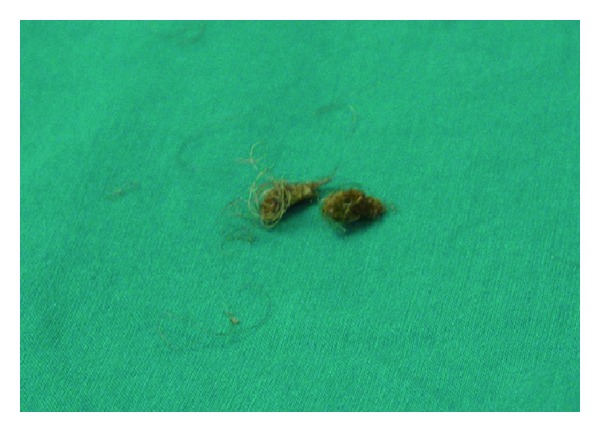
Case 2: removed hairballs.

**Figure 5:**
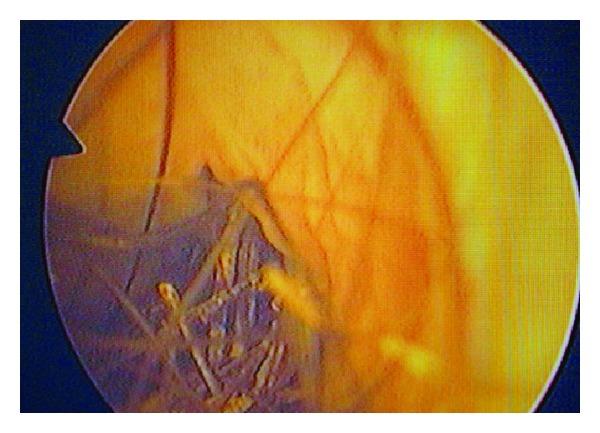
Case 2: initial urethroscopic appearance.

The patient underwent outpatient urethrocystoscopy that revealed two hairballs with concurrent microlithiasis within small diverticula of the bulbar urethra. These two hairballs were removed with a semirigid grasper (Figure 4). Hairs and hair follicles were also recognized (Figure 5). The hairs were removed by plucking with grasping forceps. In order to stop hair growth on the urethra mucosa, depilating agents were instilled. The solution composed of 10 mL of a dilute solution of thioglycolate saline, 10 mL of lidocaine jelly (2%), and 1 mL (40 mg) of gentamicin. It was introduced at high pressure into the urethra by means of a syringe to overlay the whole urethra. The patient was next instructed to drink plenty of fluids and to void frequently for the next hours. This instillation was repeated every 2 weeks for the first 3 months and then on monthly basis for one year without any severe complication reported from the patient. Visual assessment of the hair reduction was recorded the following months. Despite the prevention of hair growth, hair follicles were not totally destroyed. No further treatment was provided to the patient.

## 4. Discussion

The surgical correction of hypospadias is a challenging issue in pediatric urology. Well-known long-term complications include urethrocutaneous fistula, stricture, the formation of urethral diverticula, urethral hair growth, and hairball formation with concurrent microlithiasis at a rate of between 5% and 15% [1, 2]. Those can be the cause of recurring urinary tract infections (UTIs) and dysuria with incidence rates ranging from 3% to 8%, although it is probable that the overall percentage is higher [2].

In order to avoid hair growth in the neourethra, several prevention strategies have been followed [3–5]. However, they often fail and treatment of the hair-bearing urethra is required. Different options for this treatment include CO_2_ laser desiccation, YAG laser photocoagulation, grasper extraction, diode laser, electrolysis, and hair tricholysis with thioglycolate or open surgery revision [6–10].

In our first case, the management of penile urethral diverticulum by simple surgical removal was sufficient. In the second case, we were presented with a patient with urethral hair growth, dysuria, and recurring UTIs. On initial screening, his post-void residual was negligible and the reported UTIs were not attributed to residual urine in the bladder. The two hairballs with the microlithiasis that were discovered in the bulbar urethra were physically removed with a semirigid grasper, followed by local instillation of thioglycolate solution for hair tricholysis. Chemical depilation by epilating agents such as thioglycolate break the strong cross-linkage disulfide bonds in hair keratin, thus eliminating hair growth and preventing its reformation [7]. We have chosen this chemical substance taking in consideration the risk of skin allergy, irritation, and sensitization. The results of the treatment were encouraging but it did not achieve total elimination of hair follicles. So, the use of chemical depilating agents, although effective, is not a complete treatment all by itself. More recent therapies should be considered in conjunction with older established practices in order to achieve the ideal treatment.
